# Association between a rare SNP in the second intron of human Agouti related protein gene and increased BMI

**DOI:** 10.1186/1471-2350-10-63

**Published:** 2009-07-14

**Authors:** Ineta Kalnina, Ivo Kapa, Valdis Pirags, Vita Ignatovica, Helgi B Schiöth, Janis Klovins

**Affiliations:** 1 Latvian Biomedical Research and Study Centre, Riga, Latvia; 2 Department of Endocrinology, Pauls Stradins Clinical University Hospital, University of Latvia, Riga, Latvia; 3 Department of Neuroscience, Functional Pharmacology, Uppsala University, Uppsala, Sweden

## Abstract

**Background:**

The agouti related protein (AGRP) is an endogenous antagonist of the melanocortin 4 receptor and is one of the most potent orexigenic factors. The aim of the present study was to assess the genetic variability of *AGRP *gene and investigate whether the previously reported SNP rs5030980 and the rs11575892, a SNP that so far has not been studied with respect to obesity is associated with increased body mass index (BMI).

**Methods:**

We determined the complete sequence of the *AGRP *gene and upstream promoter region in 95 patients with severe obesity (BMI > 35 kg/m^2^). Three polymorphisms were identified: silent mutation c.123G>A (rs34123523) in the second exon, non-synonymous mutation c.199G>A (rs5030980) and c.131-42C>T (rs11575892) located in the second intron. We further screened rs11575892 in a selected group of 1135 and rs5030980 in group of 789 participants from the Genome Database of Latvian Population and Latvian State Research Program Database.

**Results:**

The CT heterozygotes of rs11575892 had significantly higher mean BMI value (p = 0.027). After adjustment for age, gender and other significant non-genetic factors (presence of diseases), the BMI levels remained significantly higher in carriers of the rs11575892 T allele (p = 0.001). The adjusted mean BMI value of CC genotype was 27.92 ± 1.01 kg/m^2 ^(mean, SE) as compared to 30.97 ± 1.03 kg/m^2 ^for the CT genotype. No association was found between rs5030980 and BMI.

**Conclusion:**

This study presents an association of rare allele of *AGRP *polymorphism in heterozygous state with increased BMI. The possible functional effects of this polymorphism are unclear but may relate to splicing defects.

## Background

The central melanocortin system and its components play an important role in regulation of food intake and energy balance and it is known as a major pathway of leptin signaling (reviewed in [1,2]). Agouti related protein (AGRP) is predominantly located in ARC neurons that co-express neuropeptide Y (NPY), also a strong mediator of regulation of energy balance. *AGRP *is evolutionary conserved gene [3] that acts as endogenous melanocortin 4 receptor (MC4R) and melanocortin 3 receptor (MC3R) antagonist (inverse agonist) with strong orexigenic effect (for review see [4]). Both ICV injection [5,6] and transgenic overexpression [7] of AGRP leads to increased food consumption and obesity in mice. Elevated plasma levels of AGRP have been reported in obese men [8] in comparison to non-obese men. In addition, AGRP levels are increased in humans as a result of fasting [9], indicating a possible role of AGRP in periphery.

The *AGRP *gene is located on chromosome 16q22, consists of four exons and expresses two alternatively spliced variants that differ in presence or absence of the 5' non-coding exon. The long transcript variant is predominantly expressed in the hypothalamus while the shorter transcript lacking the 5' untranslated exon is found in peripheral tissues such as testis, lung and kidney [10,11]. A few single nucleotide polymorphisms (SNPs) have previously been identified in *AGRP *gene that are associated with energy homeostasis disorder phenotypes. Two SNPs in the promoter region -3019G>A and -38C>T have been found in individuals of African origin but not in Caucasians [12]. Both these SNPs are in complete linkage disequilibrium (LD) and are associated with leanness, decreased risk for type two diabetes (T2DM) and reduced macronutrient intake [12-14]. Another SNP in the third exon of the *AGRP *gene that changes the alanine at position 67 to a threonine (rs5030980) has been identified, and is so far, found in Caucasians only. This SNP has been shown to be associated with anorexia nervosa [15]. Homozygosity of Ala67 did not have any association to a metabolic phenotype at mean age of 25 years but it was associated with late onset obesity at mean age of 53 years [16]. Patients homozygous for the Thr67 allele had significantly reduced body fatness [17]. In the present study we demonstrate association of a C>T polymorphism at position c.131-42C>T (rs11575892) with increased BMI in cohort of patients from the Latvian Genome Database.

## Methods

### Study subjects

The study was based on data and samples from the Genome Database of Latvian Population (Biobank 1), the disease based biobank comprising 1173 subjects and 439 subjects from population based Latvian State Research Program Database (Biobank 2). Both biobanks were collected by using the same protocols, questionnaires and sample treatment techniques. Briefly; individuals were required to be over the age of 18; the health status was recorded, the diagnoses was based on approved clinical criteria according to the ICD-10 codes (International Classification of Diseases); anthropometric measurements (including weight and stature), ethnic, social, environmental information and familial health status was acquired based on self-reported questionnaire. Written informed consent was obtained from all participants. We selected 95 of the most obese (BMI > 35 kg/m^2^) subjects from the database for initial screening using sequencing. The study group for the subsequent genotyping of rs11575892, was selected from all available subjects with validated health records and questionnaires in biobank by exclusion of patients with diseases whose outcome or treatment may influence BMI, including all types of cancer and diseases of thyroid gland, but not excluding patients with cardiovascular diseases. Patients with metabolic diseases were included in the study as well. In total 1135 subjects were selected based on these criteria. This sample group included 73 individuals from the 95 that were sequenced. rs5030980 was genotyped subsequently in total of 789 samples comprising 350 samples from Biobank 1 and all 439 samples included in study from Biobank 2. The 348 samples from initial selection were not available for genotyping of rs5030980. Study protocol was approved by Central Medical Ethics Committee of Latvia.

### Genetic analysis

The *AGRP *gene containing genomic DNA region including 5' upstream region and the entire coding region (positions from -1102 bp to +1027 respective to start codon) were amplified in two individual PCRs. Both strands of amplification products were directly sequenced using set of six primers (primer sequences and reaction conditions can be provided upon request from authors). In total 95 overweight individuals (BMI > 35 kg/m^2^) were sequenced with 100% success rate. All chromatograms were manually inspected using Contig Express software of Vector NTI Advance 9.0 package. Presence of polymorphisms was confirmed by opposite strand analysis. Restriction enzyme fragment polymorphism analysis of rs11575892 was used for genotyping the 698 samples from Biobank 1. Primer with a sequence mismatch in 3' part; 5'-CCCTCCCCTGGGAGGTGGG AG-3' (mismatched position underlined, wild type cuts only) was used to create mutation conditional AluI restriction sites in PCR reaction with reverse primer; 5'-GGTGAGGGAGTTTGGTGCTGG-3'. The amplified PCR product contained additional wt AluI site allowing to control the restriction reaction for false positive results. The AluI restriction products were visualized on PAAG gels. The samples with polymorphisms were verified by direct sequencing. Genotyping of the rs11575892 in all other samples and genotyping of rs5030980 was performed using pre-designed TaqMan SNP Genotyping Assays (Applied Biosystems, Foster City, California, USA) on ABI 7500 Real-Time PCR system (Applied Biosystems) according to the supplier's recommendations. In addition, the 5' upstream region of the *AGRP *sequence for all carriers of the rs11575892 from Biobank 1 was determined by sequencing. The designations of the SNP's are based on recommendation by the Human Genome Variation Society.

### Statistical analysis

Statistical analyses were performed with the statistical package SPSS (Standard Version 12.0.0;SPSS, Chicago, IL, USA). In contrast to the natural BMI values, the logarithmically transformed BMI values displayed normal distribution and were further used for all quantitative analysis. A one-way ANOVA was used in univariate analysis to test for association of log-BMI values with other categorical variables. Linear regression analysis was used to study relationship between log-BMI and age. General linear model (GLM) approach was applied to study association between each SNP and log-BMI adjusting for main effects of age, gender and those factors that showed association with logBMI under separate ANOVA tests (presence of hypertension, angina pectoris, myocardial infarction, heart failure, dyslipidemia and T2DM). Adjusted mean logBMI values, corresponding CI intervals and significance of association were estimated according to the GLM model. Logistic regression analysis adjusting for age and gender was used for dichotomous trait analysis. Power calculations were performed using Quanto v.1.2.3 [18]. Our sample size provided 80% power (at α = 0.05) to detect difference between rs11575892 genotypes in logBMI of >0.040 assuming minor allele frequency of 0.015 and to detect difference between rs5030980 genotypes in logBMI of >0.028 assuming minor allele frequency of 0.044. PLINK 1.00 software [19] was used to perform Hardy-Weinberg test, LD calculations, haplotype based association and permutation test for ANOVA and multivariate linear regression using label-swapping between SNP and logBMI values but leaving intact correlation with other covariates. 100000 permutations were performed for each analysis and we used corrected (EMP2) p-values. These values are corrected based on calculation of the proportion of permutations in which any of the test statistics exceeds the particular observed statistic and are more stringent then uncorrected p-values.

## Results and Discussion

Direct sequencing of the coding and 5' untranslated regions of the human *AGRP *gene in 95 obese patients with BMI > 35 kg/m^2 ^revealed three polymorphisms: silent mutation c.123G>A (rs34123523) in second exon (minor allele frequency MAF = 0.042), non-synonymous mutation c.199G>A (rs5030980) in third exon (MAF = 0.042) and c.131-42C>T (rs11575892) located in second intron (MAF = 0.016). As reported previously [15] rs34123523 and rs5030980 were in complete linkage disequilibrium (LD) with each other (D' = 1.00, r^2 ^= 1.00) while none of these subjects with both SNPs had rs11575892. The allele frequency of rs5030980 was in agreement (MAF from 0.036 to 0.05) with previous reports [13,20]. None of the SNPs currently present in the SNP databases or any new SNP were found in the 5' untranslated region of the *AGRP *gene. The SNPs reported for this region are however with low frequency (MAF = 0.005 for rs34731556 and rs34018897) or found in subjects of African origin (rs5030981), that may explain the low number of SNPs in our study.

The rs11575892 had not previously been associated with any phenotype and we tested the association between this SNP and BMI in a cohort of 1135 individuals from the biobank collections that were available to us. Characteristics of the study population for all subjects are presented in Table 1. The estimated minor allele frequency of rs11575892 was 0.015 and no significant departure from Hardy-Weinberg equilibrium was observed (p = 1.0). When tested for non-genetic factors, we found that hypertension, angina pectoris, heart failure, myocardial infarction, type 2 diabetes (T2DM) and dyslipidemia were significantly (p < 0.05) associated with increased logBMI. We did not find significant difference in logBMI between females and males (p = 0.34). The results of association between non-genetic factors and BMI are displayed in Additional File 1. The age of the patients correlated significantly with increased BMI (r = 0.204; P < 0.001), when analyzed using linear regression. In order to remove bias from confounding factors, we adjusted association of rs11575892 with BMI using general linear model including only those factors and covariate that showed significant association with BMI (Table 2). rs11575892 CT heterozygotes displayed significantly higher (p = 0.001) mean BMI value compared to the CC genotype (Table 2). To minimize the false-positive results given the relatively small sample size and SNP frequency, we repeated the above mentioned analysis performing permutation test with 'label swapping' method for both analysis using exactly the same parameters. Corrected empirical P values generated by permutation test were significant (p = 0.0016) in multivariate linear regression analysis. We also used median BMI (27.34 kg/m^2^) as a cut off value for overweight threshold in a categorical analysis. A significant difference in genotype distribution between the normal and the overweight groups was found in logistic regression analysis adjusting for age and sex (p = 0.011) (Table 2).

**Table 1:** Characteristics of the study population.

**Variable**	**Number**	**Percentage**	**Mean ± SD**
Total number	1135	-	-
Females	613	54.0	-
Males	522	46.0	-
Patients with:			
Hypertension	375	33.0	-
*Angina pectoris*	381	33.6	-
Myocardial infarction	246	21.7	-
Heart failure	292	25.7	-
Atrial fibrillation	24	2.1	-
T1DM	53	4.7	-
T2DM	146	12.9	-
dyslipidemia	136	12.0	-
Age (years)	1135	-	56.68 ± 12.52
BMI (kg/m^2^)	1135	-	28.07 ± 5.36
logBMI	1135	-	1.4407 ± 0.0804

T1DM, type 1 diabetes; T2DM, type 2 diabetes

SD, standard deviation; BMI, body mass index

**Table 2:** Association of BMI with two SNPs

GLM analysis^a^					
SNP					P value

rs11575892	Genotype	C/C	C/T	T/T	0.001
	Number	1101	34	-	
	Mean logBMI ± SE	1.446 ± 0.005	1.491 ± 0.014	-	
	(95% CI)	(1.436–1.456)	(1.463–1.518)	-	
	Mean BMI^c ^(kg/m2)	27.92	30.97	-	

rs5030980	Genotype	G/G	G/A	A/A	0.986
	Number	720	68	1	
	Mean logBMI ± SE	1.458 ± 0.007	1.458 ± 0.011	1.471 ± 0.076	
	(95% CI)	(1.445–1.472)	(1.436–1.481)	(1.322–1.620)	
	Mean BMI^c ^(kg/m2)	28.70	28.70	29.58	

Categorical analysis ^b^					

SNP		Genotype distribution (n)		OR (95% CI)	P value
		BMI <= median	BMI > median		

rs11575892	C/C	558	543	2.59 (1.24–5.37)	0.011
	C/T	12	22		

rs5030980	G/G	368	352	1.08 (0.65–1.78)	0.767
	G/A	33	35		
	A/A	0	1		

SE, standard error of mean; CI, confidence interval; OR, odds ratio.

a- values from general linear model adjusted for gender, age, presence of hypertension, angina pectoris, myocardial infarction, heart failure and T2DM

b- values from logistic regression adjusted for age and gender

c- BMI values back-transformed from mean logBMI values

These results indicate that association of rs11575892 with increased BMI is not due to possible confounding by specific diseases or other factors in study cohort. Nevertheless we cannot completely exclude the role of different environmental variables such as social background, diet and physical activity that could have impact on BMI, but such parameters were not accessible for present analysis. Due to low frequency of the rs11575892 polymorphism, we could only estimate the effect of this genetic variant in its heterozygous state, as we did not detect any homozygous individuals who may have pronounced effect of this SNP on BMI. The power analyses show that the present sample size had >80% power to detect changes in our logBMI of 0.045. Since rs11575892 has not previously been studied with respect to obesity and no association for this polymorphism has been found with anorexia by Vink et al[15], further studies with increased sample size or case-control studies with stratification for environmental factors such as social background, diet and physical activity would be valuable to verify the effects of rs1157892. There are reports that show effects of other *AGRP *polymorphisms on body weight are expressed in age dependant fashion [16]. This study group consists mostly of elderly people (mean 59.0 years) and this could contribute to enhance the effect of this particular SNP on BMI.

In order to test previously reported associations of rs5030980 with decreased body fatness we subsequently performed genotyping in available samples (n = 789) that were used for genotyping of rs1157892. The estimated minor allele frequency of rs11575892 was 0.044 and we did not observe significant departure from Hardy-Weinberg equilibrium (p = 1.0). Pairwise LD analysis showed absence of LD between both SNPs (D' = 1.00, r^2 ^= 0.001). No association of rs5030980 with BMI was found neither in GLM or logistic regression analysis (Table 2). We did not find any difference in mean values between GG and GA genotypes as opposite to previous report [16]. We found only one AA genotype carrier in our study group with slightly increased BMI value (29.58 kg/m^2^). This is in disagreement with results of Marks et.al where carriers of AA displayed significantly lower BMI [17]. Relatively low frequency of this SNP however permits to compare these effects with sufficient power. Haplotype analysis did not return significant difference in BMI values between three possible haplotypes (data not shown).

**Figure 1:**
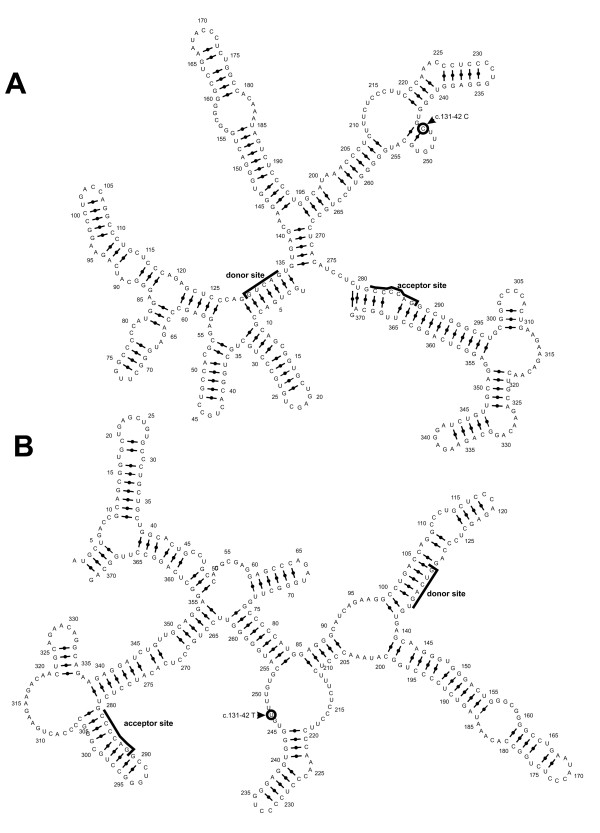
**Secondary RNA structure model of AGRP gene fragments containing either C (A) or T (B) at position c.131-42**. Donor and acceptor sites of second AGRP intron are marked with bold line. Nucleotide positions corresponding to rs1157892 are circled. Modeling was done using Mfold program from Vienna RNA package .

The previously studied SNPs in the promoter region and the coding sequence of *AGRP *have been considered to be functional mutations [21-23], while functional importance of rs1157892 is unclear. Analysis of secondary RNA structure models of this region shows significant change when this SNP is present (see Figure 1) that could influence a splicing efficiency or promote an alternative splicing. However, if rs1157892 induces production of truncated or defective AGRP peptide, it would result in lower stimulation of food intake and lower BMI what is opposite to the effects found in our study. Our findings may however be explained by action of the N-terminal part of AGRP. It has recently been shown that AGRP is posttranslationally cleaved after Arg82 [24] and it is only the C-terminal peptide that acts on the melanocortin receptors. Even though the ICV injection of N-terminal parts of AGRP (aa25-51) and (aa54-82) did not affect food consumption in rats, it caused increase of body weight and epididymal/mesenteric fat weight [25]. It is therefore possible that rs1157892 may cause splicing defects resulting in an increased fraction of the N-terminal AGRP peptide subsequently affecting body weight. Alternatively the polymorphism may promote the formation of more active AGRP isoform as a result of modified splicing reaction. We also performed screening of *AGRP *gene sequence for putative gene regulatory factor and microRNA (miRNA) binding sites using online RegRNA software (available at ). This search revealed a sequence that overlaps rs1157892 and is highly complementary to human miR-330. This complementarily is significantly decreased in presence of rs1157892. Even though most of the miRNA functions so far have been assigned to regulation at mature RNA level it is shown that miRNA can act in nucleus regulating gene expression [26]. We can thus speculate that if role of miR-330 is to suppress *AGRP *expression, the presence of rs1157892 could lead to increase of AGRP synthesis and subsequent weight gain. It is also possible that rs1157892 may be in linkage disequilibrium with other functional mutations in regulatory regions of *AGRP *gene or even in neighboring genes. It should be noted however that no polymorphisms where found in the 5' untranslated region, 1102 bp upstream of *AGRP *gene neither in carriers of rs1157892 or other samples included in sequencing.

## Conclusion

In summary, our results show novel association of a SNP in the second intron of *AGRP *with increased BMI suggesting that naturally occurring mutations in *AGRP *may be associated with enhanced effect of this peptide in the human population. This SNP could provide valuable information on the regulation of *AGRP *gene.

## Competing interests

The authors declare that they have no competing interests.

## Authors' contributions

IvK and InK carried out genetic studies, participated in statistical analysis and drafted the paper, VP supervised clinical part of the study and performed clinical characterization, VI participated in genetic studies and performed statistical analysis, HBS participated in study design and has been involved in the approval of the final version, JK coordinated the study, participated in drafting and gave final approval of version to be published.

## Pre-publication history

The pre-publication history for this paper can be accessed here:



## Supplementary Material

Additional file 1 **Table. Association between BMI and non-genetic factors**. The data provided represent the results of ANOVA analysis between BMI and other factors used in the study. Click here for file

## References

[B1] Hillebrand JJ, Kas MJ, Adan RA (2006). To eat or not to eat; regulation by the melanocortin system. Physiol Behav.

[B2] Seeley RJ, Drazen DL, Clegg DJ (2004). The critical role of the melanocortin system in the control of energy balance. Annu Rev Nutr.

[B3] Klovins J, Haitina T, Fridmanis D, Kilianova Z, Kapa I, Fredriksson R, Gallo-Payet N, Schioth HB (2004). The melanocortin system in Fugu: determination of POMC/AGRP/MCR gene repertoire and synteny, as well as pharmacology and anatomical distribution of the MCRs. Mol Biol Evol.

[B4] Stutz AM, Morrison CD, Argyropoulos G (2005). The Agouti-related protein and its role in energy homeostasis. Peptides.

[B5] Rossi M, Kim MS, Morgan DG, Small CJ, Edwards CM, Sunter D, Abusnana S, Goldstone AP, Russell SH, Stanley SA (1998). A C-terminal fragment of Agouti-related protein increases feeding and antagonizes the effect of alpha-melanocyte stimulating hormone in vivo. Endocrinology.

[B6] Lu XY, Nicholson JR, Akil H, Watson SJ (2001). Time course of short-term and long-term orexigenic effects of Agouti-related protein (86–132). Neuroreport.

[B7] Graham M, Shutter JR, Sarmiento U, Sarosi I, Stark KL (1997). Overexpression of Agrt leads to obesity in transgenic mice. Nat Genet.

[B8] Katsuki A, Sumida Y, Gabazza EC, Murashima S, Tanaka T, Furuta M, Araki-Sasaki R, Hori Y, Nakatani K, Yano Y (2001). Plasma levels of agouti-related protein are increased in obese men. J Clin Endocrinol Metab.

[B9] Hoggard N, Johnstone AM, Faber P, Gibney ER, Elia M, Lobley G, Rayner V, Horgan G, Hunter L, Bashir S (2004). Plasma concentrations of alpha-MSH, AgRP and leptin in lean and obese men and their relationship to differing states of energy balance perturbation. Clin Endocrinol (Oxf).

[B10] Ollmann MM, Wilson BD, Yang YK, Kerns JA, Chen Y, Gantz I, Barsh GS (1997). Antagonism of central melanocortin receptors in vitro and in vivo by agouti-related protein. Science.

[B11] Shutter JR, Graham M, Kinsey AC, Scully S, Luthy R, Stark KL (1997). Hypothalamic expression of ART, a novel gene related to agouti, is up-regulated in obese and diabetic mutant mice. Genes Dev.

[B12] Mayfield DK, Brown AM, Page GP, Garvey WT, Shriver MD, Argyropoulos G (2001). A role for the Agouti-Related Protein promoter in obesity and type 2 diabetes. Biochem Biophys Res Commun.

[B13] Loos RJ, Rankinen T, Rice T, Rao DC, Leon AS, Skinner JS, Bouchard C, Argyropoulos G (2005). Two ethnic-specific polymorphisms in the human Agouti-related protein gene are associated with macronutrient intake. Am J Clin Nutr.

[B14] Bonilla C, Panguluri RK, Taliaferro-Smith L, Argyropoulos G, Chen G, Adeyemo AA, Amoah A, Owusu S, Acheampong J, Agyenim-Boateng K (2006). Agouti-related protein promoter variant associated with leanness and decreased risk for diabetes in West Africans. Int J Obes (Lond).

[B15] Vink T, Hinney A, van Elburg AA, van Goozen SH, Sandkuijl LA, Sinke RJ, Herpertz-Dahlmann BM, Hebebrand J, Remschmidt H, van Engeland H (2001). Association between an agouti-related protein gene polymorphism and anorexia nervosa. Mol Psychiatry.

[B16] Argyropoulos G, Rankinen T, Neufeld DR, Rice T, Province MA, Leon AS, Skinner JS, Wilmore JH, Rao DC, Bouchard C (2002). A polymorphism in the human agouti-related protein is associated with late-onset obesity. J Clin Endocrinol Metab.

[B17] Marks DL, Boucher N, Lanouette CM, Perusse L, Brookhart G, Comuzzie AG, Chagnon YC, Cone RD (2004). Ala67Thr polymorphism in the Agouti-related peptide gene is associated with inherited leanness in humans. Am J Med Genet A.

[B18] Gauderman W, Morrison J (2006). QUANTO 1.1: A computer program for power and sample size calculations for genetic-epidemiology studies. http://hydra.usc.edu/gxe.

[B19] Purcell S, Neale B, Todd-Brown K, Thomas L, Ferreira MA, Bender D, Maller J, Sklar P, de Bakker PI, Daly MJ (2007). PLINK: a tool set for whole-genome association and population-based linkage analyses. Am J Hum Genet.

[B20] van Rossum CT, Pijl H, Adan RA, Hoebee B, Seidell JC (2006). Polymorphisms in the NPY and AGRP genes and body fatness in Dutch adults. Int J Obes (Lond).

[B21] Bai F, Rankinen T, Charbonneau C, Belsham DD, Rao DC, Bouchard C, Argyropoulos G (2004). Functional dimorphism of two hAgRP promoter SNPs in linkage disequilibrium. J Med Genet.

[B22] Sozen MA, de Jonge LH, Greenway F, Ravussin E, Smith SR, Argyropoulos G (2007). A rare mutation in AgRP, +79G>A, affects promoter activity. Eur J Clin Nutr.

[B23] de Rijke CE, Jackson PJ, Garner KM, van Rozen RJ, Douglas NR, Kas MJ, Millhauser GL, Adan RA (2005). Functional analysis of the Ala67Thr polymorphism in agouti related protein associated with anorexia nervosa and leanness. Biochem Pharmacol.

[B24] Creemers JW, Pritchard LE, Gyte A, Le Rouzic P, Meulemans S, Wardlaw SL, Zhu X, Steiner DF, Davies N, Armstrong D (2006). Agouti-related protein is posttranslationally cleaved by proprotein convertase 1 to generate agouti-related protein (AGRP)83-132: interaction between AGRP83-132 and melanocortin receptors cannot be influenced by syndecan-3. Endocrinology.

[B25] Goto K, Inui A, Takimoto Y, Yuzuriha H, Asakawa A, Kawamura Y, Tsuji H, Takahara Y, Takeyama C, Katsuura G (2003). Acute intracerebroventricular administration of either carboxyl-terminal or amino-terminal fragments of agouti-related peptide produces a long-term decrease in energy expenditure in rats. Int J Mol Med.

[B26] Place RF, Li LC, Pookot D, Noonan EJ, Dahiya R (2008). MicroRNA-373 induces expression of genes with complementary promoter sequences. Proc Natl Acad Sci USA.

