# Supplementary material for: Association between a rare SNP in the second intron of human Agouti related protein gene and increased BMI
Source: BMC Med Genet. 2009 Jul 14;10:63. doi: 10.1186/1471-2350-10-63 (PMC2714840; doi:10.1186/1471-2350-10-63)
Supplement: Additional file 1 — Table. Association between BMI and non-genetic factors. The data provided represent the results of ANOVA analysis between BMI and other factors used in the study. [file 1471-2350-10-63-S1.doc]

**Supplementary table: Association between BMI and non-genetic factors**

| **Characteristic** | | | | **Mean logBMI**  **(SE)** | | | **Mean BMI ^a^**  **(kg/m^2^)** | | **P-value^b^** |
| --- | --- | --- | --- | --- | --- | --- | --- | --- | --- |
| Gender | | | |  | | |  | |  |
|  | Male  Female | | | 1.438 (0.003)  1.443 (0.004) | | | 27.41  27.73 | | 0.342 |
| Hypertension | | | |  | |  | |  | |
|  | | No  Yes | | 1.428 (0.003)  1.468 (0.004) | | | 26.79  29.37 | | <0.001 |
| *Angina pectoris* | | |  | | |  | |  | |
|  | | No  Yes | | 1.436 (0.003)  1.451 (0.004) | | | 27.28  28.24 | | 0.002 |
| **Myocardial infarction** | | | | |  |  | |  | |
|  | | No  Yes | | 1.438 (0.003)  1.452 (0.004) | | | 27.41  28.31 | | 0.011 |
| Heart failure | | |  | | |  | |  | |
|  | | No  Yes | | 1.435 (0.003)  1.457 (0.004) | | | 27.22  28.31 | | <0.001 |
| **Atrial fibrillation** | | |  | | |  | |  | |
|  | | No  Yes | | 1.442 (0.002)  1.466 (0.010) | | | 28.01  29.26 | | 0.112 |
| T1DM | | |  | | |  | |  | |
|  | | No  Yes | | 1.441 (0.002)  1.424 (0.010) | | | 27.61  26.54 | | 0.120 |
| T2DM | | |  | | |  | |  | |
|  | | No  Yes | | 1.433 (0.002)  1.492 (0.007) | | | 27.10  31.04 | | <0.001 |
| Dyslipidemia | | |  | | |  | |  | |
|  | | No  Yes | | 1.439 (0.003)  1.457 (0.006) | | | 27.47  28.64 | | 0.023 |

T1DM, type 1 diabetes; T2DM, type 2 diabetes; SE, standard error of mean; BMI, body mass index (kg/m^2^)

a- BMI values back-transformed from logBMI

b-p values based on logBMI comparison
